# Tendência Temporal da Mortalidade por Doenças Isquêmicas do Coração no Nordeste Brasileiro (1996–2016): Uma Análise Segundo Gênero e Faixa Etária

**DOI:** 10.36660/abc.20200222

**Published:** 2021-07-15

**Authors:** Gibson Barros de Almeida Santana, Thiago Cavalcanti Leal, João Paulo Silva de Paiva, Leonardo Feitosa da Silva, Lucas Gomes Santos, Tatiana Farias de Oliveira, Rodrigo da Rosa Mesquita, Jéssica Alves Gomes, Carlos Dornels Freire de Souza, Amanda Karine Barros Ferreira Rodrigues

**Affiliations:** 1 Universidade Federal de AlagoasMaceióALBrasilUniversidade Federal de Alagoas, Maceió, AL - Brasil

**Keywords:** Isquemia Miocárdica/mortalidade, Epidemiologia, Fatores Socioeconômicos, Mortes, Análise Estatística, Demografia, Saúde Pública

## Abstract

**Fundamentos:**

As doenças isquêmicas do coração (DIC) são a primeira causa de mortes dentre as doenças cardiovasculares (DCV).

**Objetivo:**

Descrever o perfil sociodemográfico e analisar tendência da taxa de mortalidade por DIC, segundo sexo e por faixa etária, nos estados da região Nordeste do Brasil, 1996-2016.

**Métodos:**

Estudo ecológico envolvendo a mortalidade por DIC nos estados do Nordeste. Variáveis analisadas: sexo, idade, escolaridade, estado civil, categoria do CID-10 e estado de residências. Foram calculadas taxas brutas e padronizadas. Os dados de óbitos foram coletados do Sistema de Informações sobre Mortalidade; e os dados populacionais, do Instituto Brasileiro de Geografia e Estatística (IBGE). Nas análises temporais, utilizou-se o modelo de regressão por pontos de inflexão, com cálculo do percentual de variação anual (APC,* Annual Percent Change*) e do percentual de variação médio do período (AAPC,* Average Annual Percent Change*). Considerou-se intervalo de confiança de 95% e significância de 5%.

**Resultados:**

Registrou-se 405.916 óbitos por DIC na região Nordeste durante o período estudado. O perfil de óbitos caracteriza-se por homens (n=229.006; 56,42%), idosos (n=301.379; 74,25%), raça/cor parda (n=197.936; 48,76%), fundamental ou <4 anos na escola (n=232.599; 57,30%) e casados (n=179.599; 44,25%). Houve destaque incomum para o aumento na taxa de incremento anual na faixa etária de adolescentes (AAPC: 5,2%, p<0,01). A taxa de mortalidade regional padronizada cresceu de 30,7/100 mil habitantes, em 1996, para 53,8/100 mil, em 2016 (AAPC:2,8%; p<0,01). Todos os nove estados apresentaram tendência estatisticamente significante de crescimento, com ênfases para o Maranhão (AAPC:7,6%; p<0,01) e o Piauí (AAPC:6,0%; p<0,01).

**Conclusão:**

O perfil prevalente observado foi de homens, idosos, raça/cor parda, baixa escolaridade e casados. A mortalidade por DIC apresentou tendência de crescimento em todos os estados, ainda que com padrão desigual entre as unidades federadas.

## Introdução

O rápido envelhecimento populacional, o processo de urbanização acelerado e as mudanças socioeconômicas contínuas impactaram no estilo de vida dos indivíduos nas últimas décadas, modificando o perfil epidemiológico.^1-3^Nesse cenário, as doenças crônicas não transmissíveis (DCNT) – doenças cardiovasculares (DCV), cânceres, doenças respiratórias crônicas e diabetes melito (DM) – ganharam espaço no contexto epidemiológico e social, constituindo um problema global de saúde responsável por 40 milhões de mortes anuais, sendo 17 milhões em decorrência de DCV.^4^

No Brasil, em 2016, as DCNT resultaram em 707 mil óbitos, dos quais 362 mil foram por DCV.^5^ Em virtude da dimensão continental do país, a dinâmica da mortalidade por DCNT entre as regiões e unidades da federação brasileira não ocorre de modo homogêneo. São Paulo, por exemplo, segue o padrão dos países desenvolvidos, com redução da taxa de mortalidade,^6^ enquanto na região Nordeste, sob contexto socioeconômico diferente (piores indicadores socioeconômicos e difícil acesso à saúde), o cenário é o oposto.^7,8^

Dentre as DCV, as mais prevalentes são as doenças isquêmicas do coração (DIC), responsáveis por 116 mil óbitos no Brasil somente no ano de 2016.^5^ As DIC apresentam-se como um fluxo sanguíneo insuficiente e suprimento de oxigênio inadequado ao coração, cujas consequências microscópicas serão: lesão isquêmica do miocárdio, dano irreversível aos cardiomiócitos e hipertrofia de sobrecarga em áreas não necróticas.^9^ De forma pragmática, os indivíduos apresentam aumento na dependência das atividades de vida diária e de mobilidade, ou seja, há diminuição da capacidade funcional.^10^

Os fatores de risco dessas doenças são classificados em duas categorias: modificáveis e não modificáveis. Os fatores modificáveis são hipertensão arterial sistêmica (HAS), obesidade, sedentarismo, hábitos alimentares inadequados, tabagismo, consumo de bebidas alcoólicas, dislipidemias e resistência à insulina; os não modificáveis referem-se a idade, gênero, raça e hereditariedade.^11,12^ O controle dos fatores de risco modificáveis reduz em larga escala a morbimortalidade pelas doenças cardiovasculares.^13^

A realização de investigações sobre a evolução temporal da mortalidade por DCV no Nordeste é fundamental para a tomada de decisão em saúde, já que podem contribuir para a definição de áreas prioritárias de intervenção e para o desenvolvimento de estratégias e ações voltadas para a melhoria de saúde da população, sobretudo no que diz respeito aos fatores de risco.^14^

A região Nordeste carece de estudos descritivos pormenorizados sobre suas características epidemiológicas. Com base no exposto, o presente estudo objetivou descrever o perfil sociodemográfico e analisar tendência da taxa de mortalidade por DIC, segundo sexo e por faixa etária, nos estados da região Nordeste do Brasil, de 1996 a 2016. Dessa forma, amplia-se o leque científico acerca de sua situação em saúde e os determinantes sociais que a compõem.

## Métodos

### Desenho de estudo e fonte de dados

Trata-se de estudo de séries temporais envolvendo todos os óbitos por DIC ocorridos no Nordeste brasileiro, no período de 1996 a 2016. Os registros dos óbitos foram obtidos do Sistema de Informações sobre Mortalidade (SIM) do Departamento de Informática do Sistema Único de Saúde do Ministério da Saúde (http://www.datasus.gov.br/). No processo de coleta, foram considerados os códigos I20 a I25 da Classificação Internacional de Doenças (CID-10): I20 – angina *pectoris*; I21 – infarto agudo do miocárdio; I22 – infarto do miocárdio recorrente; I23 – algumas complicações atuais subsequentes ao infarto agudo do miocárdio; I24 – outras doenças isquêmicas agudas do coração; I25 – doença isquêmica crônica do coração. Os dados populacionais necessários para o cálculo dos indicadores foram coletados do IBGE, censo de 2010.

### Área de estudo

A região Nordeste do Brasil é composta por nove estados (Maranhão, Piauí, Ceará, Rio Grande do Norte, Paraíba, Pernambuco, Alagoas, Sergipe e Bahia) e uma população estimada de 57 milhões de habitantes, o que corresponde a 28% da população nacional – segunda região mais populosa do Brasil.^15^

### Variáveis de estudo

Foram incluídas no estudo variáveis sociodemográficas (faixa etária, escolaridade, estado civil, cor/raça e unidades da federação), categoria da classificação internacional de doenças (CID-10) e as taxas brutas e padronizadas de mortalidade, segundo gênero e faixa etária.

Para o cálculo das taxas, foram utilizadas as seguintes equações:

- Taxa de mortalidade anual = (número de óbitos por DIC no local e ano / População residente no local e ano) x 100 mil
- Taxa de mortalidade do perído = (Média simples do número de óbitos por DIC, do período, no local / População residente no meio do período no local) x 100 mil

Para a padronização das taxas de mortalidade, adotou-se o método direto, considerando como população padrão a brasileira do ano de 2010. Foram adotadas as seguintes faixas etárias no processo de padronização: 0 a 9 anos, 10 a 19 anos, 20 a 29 anos, 30 a 39 anos, 40 a 49 anos, 50 a 59 anos e 60 anos ou mais anos.

### Tratamento estatístico

Inicialmente, as variáveis sociodemográficas foram analisadas com o emprego da estatística descritiva simples (frequências absoluta e relativa). A análise temporal foi realizada com o emprego do modelo de regressão por pontos de inflexão (*joinpoint regression model*). O modelo analisa se uma linha com múltiplos segmentos é mais adequada para explicar o comportamento temporal de um conjunto de dados quando comparada com uma linha reta ou com menos segmentos. Dessa forma, a tendência de cada indicador é classificada em estacionária, crescente ou decrescente, conforme inclinação da reta de regressão. Foram calculadas a variação percentual anual (APC, *Annual Percent Change*) e a variação média do período (AAPC, *Average Annual Percent Change*).^16^

Na análise, foram adotados os seguintes parâmetros: i) mínimo de zero *joins*, ii) máximo de quatro *joins*, iii) seleção do modelo pelo teste de computação de Monte Carlo (n= 4.499 permutações), iv) método de autocorrelação dos erros baseado na data, v) intervalo de confiança de 95% (IC95%) e vi) nível de significância de 5%. Tais análises foram realizadas com o auxílio do *Joinpoint regression program* (*version* 4.5.0.1*, National Cancer Institute, Bethesda,* MD, USA).

### Aspectos éticos

O presente estudo utilizou dados secundários de domínio público, nos quais não é possível a identificação dos sujeitos, razão pela qual dispensou aprovação pelo comitê de ética em pesquisa.

## Resultados

No período de 1996 a 2016, foram registrados 405.916 óbitos por DIC na região Nordeste do Brasil. Desses óbitos, 56,42% (n=229.006) eram homens; 74,25% (n=301.379), idosos; 48,76% (n=197.936), pardos; 57,30%, (n=232.599) com ensino fundamental ou <4 anos na escola; e 44,25% (n=179.599), casados. Destacou-se a proporção de campos ignorados nas variáveis escolaridade (33,54%), estado civil (8,95%) e raça/cor (15,84%). Quanto à causa do óbito, 85,07% (n=345.329) decorreram de infarto agudo do miocárdio (I21); 46,37% (n=188.217) dos óbitos residiam nos estados de Pernambuco e da Bahia (Tabela 1).

**Tabela 1:** – Caracterização sociodemográfica e categoria CID-10 dos óbitos por doenças isquêmicas do coração (DIC) ocorridas no Nordeste brasileiro, 1996-2016

Variável	Masculino n=229006 (56,42%)		Feminino n=176766 (43,55%)		Ignorado n=144 (0,03%)		Total n=405916 (100%)	
	n	%	n	%	n	%	n	%
**Faixa etária**								
<10 anos	72	0,05	49	0,02	0	0,00	121	0,03
10 a 19	469	0,20	215	0,12	1	0,70	685	0,17
20 a 29	2197	0,96	815	0,46	0	0,00	3012	0,74
30 a 39	7086	3,09	3197	1,81	3	2,08	10286	2,53
40 a 49	19380	8,46	10763	6,09	14	9,72	30157	7,43
50 a 59	38131	16,65	21493	12,16	17	11,81	59641	14,69
60 anos ou mais	161295	70,43	139991	79,20	93	64,58	301379	74,25
Ignorado	376	0,16	243	0,14	16	11,11	635	0,16
**Cor/raça**								
Branca	58574	25,58	53120	30,05	10	6,94	111704	27,52
Preta	17345	7,57	11975	6,77	1	0,70	29321	7,22
Amarela	1062	0,46	928	0,53	2	1,39	1992	0,49
Parda	115714	50,53	82208	46,51	14	9,72	197936	48,76
Indígena	385	0,17	301	0,17	0	0,00	686	0,17
Ignorado	35926	15,69	28234	15,97	117	81,25	64277	15,84
**Escolaridade**								
Fundamental ou <4 anos na escola	129138	56,39	103426	58,51	35	24,30	232599	57,30
Médio	15866	6,93	9088	5,14	4	2,78	24958	6,15
Superior	8303	3,62	3909	2,21	0	0,00	12212	3,01
Ignorado	75699	33,06	60343	34,14	105	72,92	136147	33,54
**Estado civil**								
Solteiro	45124	19,7	41989	23,75	21	14,58	87134	21,47
Casado	125600	54,85	53953	30,52	46	31,94	179599	44,25
Viúvo	26413	11,53	58356	33,01	26	18,06	84795	20,89
Separado judicialmente	6930	3,03	3642	2,06	1	0,70	10573	2,60
Outro	5254	2,29	2228	1,27	2	1,39	7484	1,84
Ignorado	19685	8,60	16598	9,39	48	33,33	36331	8,95
**Categoria CID-10**								
I20	885	0,39	875	0,5	0	0,00	1760	0,43
I21	196621	85,86	148585	84,06	123	85,42	345329	85,07
I22	591	0,25	386	0,21	0	0,00	977	0,25
I23	3	<0,01	0	0,00	0	0,00	3	<0,01
I24	6745	2,95	6320	3,58	7	4,86	13072	3,22
I25	24161	10,55	20600	11,65	14	9,72	44775	11,03
**Estados**								
Maranhão	21847	9,54	13792	7,8	24	16,67	35663	8,79
Piauí	14539	6,35	9446	5,34	16	11,11	24001	5,91
Ceará	33500	14,63	26623	15,06	26	18,06	60149	14,82
Rio Grande do Norte	17347	7,57	13335	7,54	4	2,78	30686	7,56
Paraíba	18825	8,22	14945	8,45	11	7,64	33781	8,32
Pernambuco	59385	25,93	48909	27,67	34	23,61	108328	26,69
Alagoas	11957	5,22	9034	5,11	2	1,38	20993	5,17
Sergipe	6797	2,97	5624	3,20	5	3,47	12426	3,06
Bahia	44809	19,57	35058	19,83	22	15,28	79889	19,68

*I20 – angina pectoris; I21 – infarto agudo do miocárdio; I22 – infarto do miocárdio recorrente; I23 – algumas complicações atuais subsequentes ao infarto agudo do miocárdio; I24 – outras doenças isquêmicas agudas do coração; I25 – doença isquêmica crônica do coração.*

**Figura 1:**
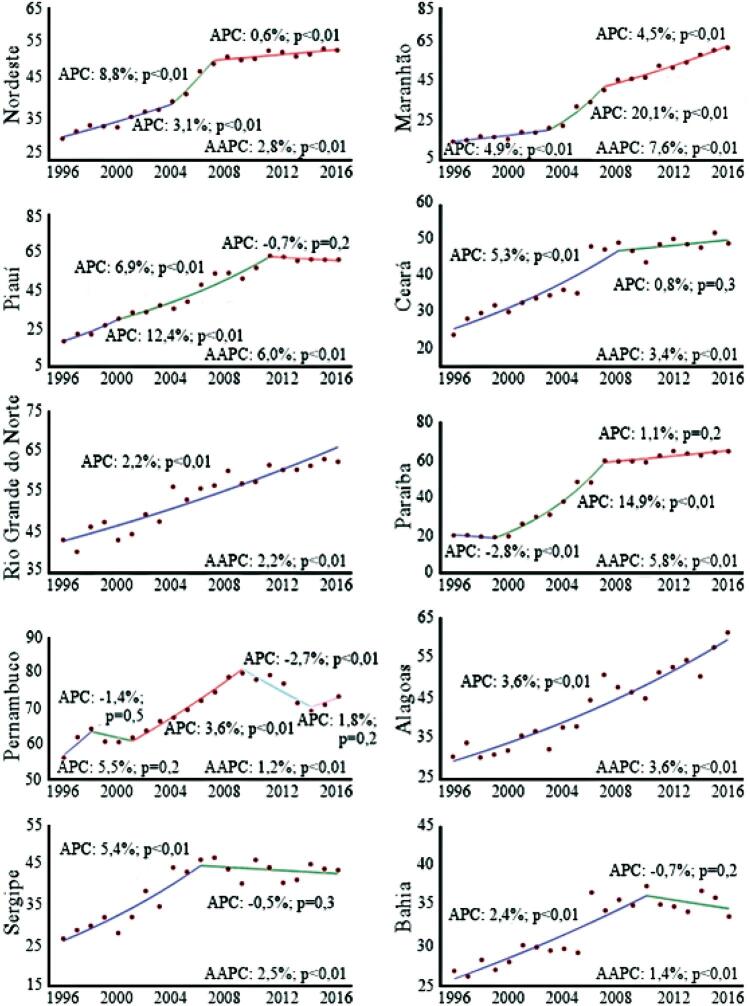
– Tendência temporal da taxa de mortalidade padronizada por doenças isquêmicas do coração (DIC), segundo estado de residência. Nordeste, Brasil, 1996-2016. APC: Annual Percent Change. AAPC: Average Annual Percent Change. * Valores de intervalo de confiança foram suprimidos da ilustração para melhor visualização e entendimento das linhas de tendência.

A taxa de mortalidade regional padronizada passou de 30,7/100 mil habitantes em 1996 para 53,8/100 mil em 2016 (AAPC 2,8%; IC95%: 1,9 a 3,7; p<0,01). Todos os nove estados apresentaram tendência significativa de crescimento, destacando-se o estado do Maranhão, cuja taxa passou de 14,8/100 mil em 1996 para 64,0/100 mil habitantes em 2016 (AAPC 7,6%; IC95%: 5,7 a 9,6; p<0,01), seguido do Piauí, no qual a taxa ascendeu de 18,7/100 mil em 1996 para 61,5/100 mil habitantes em 2016 (AAPC 6,0%; IC95%: 4,3 a 7,8; p<0,01). Pernambuco destacou-se com maior número de segmentos temporais, com quatro inflexões, cinco segmentos temporais e AAPC igual a 1,2% (IC95%: 0,0 a 2,5; p<0,01). Os estados do Rio Grande do Norte e de Alagoas apresentaram tendência linear de crescimento da mortalidade (2,2% no Rio Grande do Norte e 3,6% em Alagoas) (Figura 1).

A taxa de mortalidade média no sexo masculino (42,05/100 mil) foi 32% maior que no feminino (31,6/100 mil), ainda que com o mesmo percentual de variação médio (AAPC 4,4%; IC95%: 3,4 a 5,4; p<0,01). A faixa etária de adolescentes apresentou o maior aumento percentual médio do período (AAPC: 5,2%, p<0,01) (Tabela 2). Todos os estados apresentaram tendência de crescimento em ambos os sexos, destacando-se o Maranhão (AAPC 8,7% para o masculino e 9,3% para o feminino) e o Piauí (AAPC 7,9% para o masculino e 8,4% para o feminino) (Tabela 3).

**Tabela 2:** – Tendência temporal da taxa de mortalidade bruta por doenças isquêmicas do coração (DIC), segundo sexo e faixa etária. Nordeste, Brasil, 1996-2016

Variável	Taxa de mortalidade/100 mil			Tendência		
	1996	2016	1996-2016	Período	APC (IC95%; p valor)	AAPC (IC95%; p valor)
**Sexo**						
Masculino	25,52	60,88	42,05	1996-2004	3,7% (3,0 a 4,5; p<0,01)	4,4% (3,4 a 5,4; p<0,01)
				2004-2007	10,6% (3,4 a 18,3; p<0,01)	
				2007-2016	3,0% (2,4 a 3,6; p<0,01)	
Feminino	18,78	44,24	31,69	1996-2003	4,0% (3,1 a 5,0; p<0,01)	4,4% (3,6 a 5,2; p<0,01)
				2003-2007	9,2% (5,5 a 13,1; p<0,01)	
				2007-2016	2,6% (2,0 a 3,2; p<0,01)	
**Faixa Etária**						
0 a 9	0,01	0,10	0,05	-	-	-
10 a 19	0,21	0,39	0,31	1996-2016	5,2% (3,0 a 7,5; p<0,01)	5,2% (3,0 a 7,5; p<0,01)
20 a 29	1,00	1,96	1,42	1996-2016	2,8% (2,0 a 3,6; p<0,01)	2,8% (2,0 a 3,6; p<0,01)
30 a 39	5,79	7,18	6,66	1996-2003	-1,5% (-3,5 a 0,5; p=0,2)	0,9% (-0,8 a 2,5; p=0,4)
				2003-2007	6,8% (-1,1 a 15,4; p=0,3)	
				2007-2016	0,2% (-1,2 a 1,6; p=0,2)	
40 a 49	20,44	26,84	25,38	1996-1998	9,8% (1,1 a 19,2; p<0,01)	1,5% (0,1 a 2,9; p<0,01)
				1998-2001	-4,6% (-12,1 a 3,6; p=0,2)	
				2001-2008	4,4% (3,0 a 5,9; p<0,01)	
				2008-2016	-0,7% (-1,6 a 0,2; p=0,2)	
50 a 59	52,99	78,25	74,40	1996-2008	3,3% (2,7 a 3,9; p<0,01)	1,6% (1,1 a 2,2; p<0,01)
				2008-2016	-0,7% (-1,8 a 0,3; p=0,3)	
60 ou mais	202,03	382,28	323,15	1996-2003	3,3% (2,2 a 4,3; p<0,01)	3,3% (2,5 a 4,1; p<0,01)
				2003-2007	8,5% (4,6 a 12,6; p<0,01)	
				2007-2016	1,0% (0,4 a 1,7; p<0,01)	
População geral	22,14	52,40	36,82	1996-2004	4,0% (3,3 a 4,7; p<0,01)	4,4% (3,5 a 5,3; p<0,01)
				2004-2007	10,5% (3,9 a 17,4; p<0,01)	
				2007-2016	2,8% (2,2 a 3,4; p<0,01)	

*APC: Annual Percent Change. AAPC: Average Annual Percent Change.*

**Tabela 3:** – Percentual de variação médio (AAPC) da taxa de mortalidade por doenças isquêmicas do coração (DIC), segundo sexo e estado de residência. Nordeste, Brasil, 1996-2016

Estado	Masculino			Feminino		
	1996	2016	AAPC (IC95%; p valor)	1996	2016	AAPC (IC95%; p valor)
Maranhão	12,06	62,75	8,7% (7,0 a 10,5; p<0,01)	6,20	38,89	9,3% (7,1 a 11,7; p<0,01)
Piauí	16,16	75,49	7,9% (4,4 a 11,5; p<0,01)	9,98	45,16	8,4% (7,3 a 9,5; p<0,01)
Ceará	20,65	56,89	5,1% (4,5 a 5,8; p<0,01)	15,55	41,70	5,0% (3,9 a 5,1; p<0,01)
Rio Grande do Norte	39,61	74,56	3,5% (3,1 a 3,9; p<0,01)	27,88	53,24	3,9% (3,4 a 4,4; p<0,01)
Paraíba	20,88	78,08	6,8% (5,5 a 8,1; p<0,01)	15,53	60,00	7,1% (5,2 a 9,1; p<0,01)
Pernambuco	49,59	85,82	2,4% (1,5 a 3,3; p<0,01)	36,93	64,36	2,3% (1,3 a 3,3; p<0,01)
Alagoas	23,73	61,02	4,7% (3,1 a 6,3; p<0,01)	15,95	47,96	5,3% (4,5 a 6,1; p<0,01)
Sergipe	19,25	42,47	4,4% (3,1 a 5,7; p<0,01)	18,58	35,80	2,7% (0,5 a 4,9; p<0,01)
Bahia	21,35	39,39	3,6% (3,2 a 4,1; p<0,01)	16,32	29,05	3,6% (2,7 a 4,4; p<0,01)

*APC: Annual Percent Change. AAPC: Average Annual Percent Change.*

Na estratificação segundo faixa etária, o modelo de regressão apontou tendência de crescimento da mortalidade em todos os segmentos etários, com exceção do grupo com idade entre 30 e 39 anos (AAPC 0,9%; IC95%: -0,8 a 2,5; p=0,4) (Tabela 2). A taxa de mortalidade regional em indivíduos com 60 anos ou mais passou de 202,0/100 mil em 1996 para 382,3/100 mil habitantes em 2016, com crescimento médio anual de 3,3% (IC95%: 2,5 a 4,1; p<0,01). A faixa etária de 60 anos ou mais foi a única com crescimento estatisticamente significativo em todos os estados, destacando-se os estados do Maranhão (AAPC 8,4%; IC95%: 6,3 a 10,5; p<0,01) e do Piauí (AAPC 6,5%; IC 95%: 5,3 a 7,7; p<0,01) (Tabela 4).

**Tabela 4:** – Percentual de variação médio (AAPC) da taxa de mortalidade bruta por doenças isquêmicas do coração (DIC), segundo faixa etária e estado de residência. Nordeste, Brasil, 1996-2016

**Faixa etária**	**Maranhão**			**Piauí**			**Ceará**		
	**Taxas**		**AAPC**	**Taxas**		**AAPC**	**Taxas**		**AAPC**
	**1996**	**2016**	**(IC95%)**	**1996**	**2016**	**(IC95%)**	**1996**	**2016**	**(IC95%)**
0 a 9	0,00	0,14	-	0,00	0,19	-	0,00	0,07	-
10 a 19	0,00	0,50	-	0,15	0,84	7,0% (2,9 a 11,3; p<0,01)	0,51	0,56	4,9% (-0,3 a 10,4; p=0,5)
20 a 29	0,12	3,25	9,0% (4,9 a 13,2; p<0,01)	0,93	3,42	6,2% (3,6 a 8,9; p<0,01)	1,16	1,39	0,7% (-0,6 a 2,1; p=0,3)
30 a 39	3,17	11,62	5,8% (4,7 a 7,0; p<0,01)	6,09	10,00	3,5% (2,0 a 5,1; p<0,01)	5,80	5,31	0,8% (-0,3 a 1,9; p=0,3)
40 a 49	10,46	35,96	6,1% (1,5 a 11,0; p<0,01)	14,71	31,10	3,5% (2,5 a 4,5; p<0,01)	15,29	20,40	2,1% (1,2 a 3,1; p<0,01)
50 a 59	29,12	90,17	5,7% (3,2 a 8,1; p<0,01)	34,61	87,61	3,5% (1,4 a 5,6; p<0,01)	38,07	66,06	1,9% (0,5 a 3,4; p<0,01)
60 +	94,19	446,37	8,4% (6,3 a 10,5; p<0,01)	114,40	432,41	6,5% (5,3 a 7,7; p<0,01)	157,12	361,26	3,9% (2,8 a 5,1; p<0,01)
**Faixa etária**	**Rio Grande do Norte**			**Paraíba**			**Pernambuco**		
	**Taxas**		**AAPC**	**Taxas**		**AAPC**	**Taxas**		**AAPC**
	**1996**	**2016**	**(IC95%)**	**1996**	**2016**	**(IC95%)**	**1996**	**2016**	**(IC95%)**
0 a 9	0,18	0,00	-	0,00	0,16	-	0,00	0,07	-
10 a 19	0,34	0,34	-	0,51	0,15	-	0,23	0,54	4,5% (0,9 a 8,3; p<0,01)
20 a 29	1,14	1,83	4,7% (0,8 a 8,7; p<0,01)	0,92	2,42	5,1% (2,7 a 7,5; p<0,01)	2,10	2,75	1,5% (-0,3 a 3,2; p=0,4)
30 a 39	7,16	7,45	2,0% (0,2 a 3,7; p<0,01)	4,12	7,30	4,7% (3,5 a 5,9; p<0,01)	9,65	9,13	-1,1% (-5,9 a 4,1; p=0,4)
40 a 49	23,52	28,02	1,1% (0,2 a 2,0; p<0,01)	14,54	34,14	4,2% (1,4 a 7,1; p<0,01)	33,08	35,25	-0,2% (-3,3 a 3,0; p=0,2)
50 a 59	69,14	88,46	1,3% (0,4 a 2,2; p<0,01)	31,84	84,49	5,7% (3,9 a 7,4; p<0,01)	98,89	114,28	0,6% (-0,8 a 2,0; p=0,2)
60 +	298,31	452,97	2,4% (1,8 a 3,0; p<0,01)	138,19	469,44	6,3% (4,8 a 7,7; p<0,01)	378,85	520,30	1,2% (0,4 a 1,9; p<0,01)
**Faixa etária**	**Alagoas**			**Sergipe**			**Bahia**		
	**Taxas**		**AAPC**	**Taxas**		**AAPC**	**Taxas**		**AAPC**
	**1996**	**2016**	**(IC95%)**	**1996**	**2016**	**(IC95%)**	**1996**	**2016**	**(IC95%)**
0 a 9	0,00	0,17	-	0,00	0,00	-	0,00	0,08	-
10 a 19	0,00	0,15	-	0,00	0,00	-	0,13	0,23	-
20 a 29	0,66	2,13	3,2% (0,7 a 5,7; p<0,01)	0,35	0,50	-2,9% (-7,1 a 1,5; p=0,4)	0,75	0,99	0,8% (-0,7 a 2,3; p=0,6)
30 a 39	6,73	5,34	-0,8% (-2,3 a 0,6; p=0,2)	5,59	4,21	1,1% (-0,9 a 3,1; p=0,3)	4,31	5,34	0,4% (-0,5 a 1,2; p=0,6)
40 a 49	31,05	36,91	0,3% (-2,2 a 2,9; p=0,2)	12,47	26,10	2,9% (-0,3 a 6,1; p=0,3)	20,10	16,87	-0,1% (-0,8 a 0,6; p=0,7)
50 a 59	61,26	95,11	1,6% (-1,6 a 4,8; p=0,3)	55,05	67,15	1,6% (0,6 a 2,7; p<0,01)	45,72	50,89	0,3% (-0,7 a 1,3; p=0,2)
60 +	182,72	428,16	4,3% (3,8 a 4,9; p<0,01)	177,36	308,36	2,9% (1,7 a 4,0; p<0,01)	177,50	236,80	2,2% (1,7 a 2,7; p<0,01)

*APC: Annual Percent Change. AAPC: Average Annual Percent Change.*

Por fim, observou-se divergência na tendência segundo faixa etária ao comparar os estados. No Piauí, por exemplo, houve crescimento em todas as faixas a partir dos 10 anos de idade; ao passo que, na Bahia, o crescimento foi observado apenas em idosos (60 anos ou mais) (Tabela 4).

## Discussão

Este trabalho analisou o perfil sociodemográfico e o comportamento temporal da mortalidade por DIC na região Nordeste do Brasil no período de 1996 a 2016. O perfil dos óbitos caracterizou-se pelo predomínio do sexo masculino, acometimento de idosos, raça/cor parda e baixa escolaridade. A análise temporal demonstrou crescimento da taxa de mortalidade na região e em todos os estados, com destaque para o Maranhão e o Piauí.

O perfil observado neste estudo está em consonância com a literatura.^1,7,17-20^ O processo de envelhecimento da população traz consigo uma ampliação dos fatores de risco para DCV, destacando-se as dislipidemias, a obesidade e a HAS.^2^ Estudo realizado no município de São Paulo aponta que a razão de chance de desenvolver DCV é maior em diabéticos (*odds ratio* [OR] 1,90), tabagistas (OR 1,49), indivíduos com sobrepeso (OR 1,57) e hipertensos (OR 2,22).^20^ Estima-se que a prevalência de HAS em idosos seja seis a oito vezes superior à de adultos jovens, justificada pelo controle pressórico deficitário, em razão da cronicidade da doença e sensibilidade e restrições na terapia farmacológica. Em estudo realizado em Goiânia/GO, 912 indivíduos com HAS foram entrevistados, 72,6% estavam em tratamento e apenas 50,8% apresentavam controle pressórico.^21^

É pertinente destacar o crescimento da mortalidade na adolescência, conforme observado nesta investigação. Nessa população, estudos apontam um amplo conjunto de fatores associados ao sobrepeso e obesidade, tais como i) ingestão excessiva de açúcares simples e gorduras, ii) consumo insuficiente de frutas e hortaliças e iii) sedentarismo, que podem resultar em mortalidade precoce.^22^ O Estudo de Riscos Cardiovasculares em Adolescentes (ERICA), de 2013 a 2014, relatou prevalência de síndrome metabólica em 3,3% dos que estavam em sobrepeso e em 21,7% dos que apresentavam obesidade no Nordeste.^23^

A importância médica de alterações no perfil lipídico está relacionada à presença de aterosclerose subclínica e à possibilidade de predizer dislipidemia em vida adulta. No ERICA, observou-se maior prevalência de baixos níveis de lipoproteína de alta densidade (HDL, high density lipoproteins) nas regiões Norte e Nordeste do Brasil.^24^ Estudo realizado em Belém/PA em 2006, envolvendo 437 crianças e adolescentes, apontou 28,8% com excesso de peso e 36% com percentual de gordura elevado. Destas, 49% apresentaram alteração no perfil lipídico, com destaque para os baixos valores de HDL.^25^ Resultados semelhantes foram observados em Porto Alegre/RS, onde ocorre um crescimento da dislipidemia secundária à obesidade, caracterizada por baixos níveis de HDL e aumento dos níveis de triglicerídeos, secundário à resistência à insulina.^26^

O risco cardiovascular também recebe influência do sexo, sendo maior na população masculina. Um estudo sobre hipertensão em Goiânia/GO, realizado em 2010, mostrou que a taxa de controle dos níveis pressóricos é menor na população masculina (44,0% em homens e 54,8% em mulheres).^21^ Em Montes Claros/MG, 62,8% dos casos de DM são mulheres, justificados pela maior procura por assistência médica por essa população, que apresenta maior e melhor adesão ao tratamento.^27^ Ademais, as mulheres tem características biológicas e comportamentais capazes de reduzir o risco de doenças cardiovasculares, destacando-se o papel protetor do estradiol no endotélio vascular, o maior acesso aos serviços de saúde e o melhor desempenho no controle de fatores risco.^17^

A influência do desenvolvimento socioeconômico e do acesso aos serviços de saúde no padrão de mortalidade tem sido evidenciada em diferentes estudos.^28-30^ Investigação realizada em 2012, nos estados do Rio de Janeiro, Rio Grande do Sul e São Paulo, mostrou correlação entre aumento do PIB *per capita* e do nível de escolaridade com a diminuição da mortalidade por DIC.^31^ No Nordeste, o Maranhão, ao mesmo tempo em que apresentou o maior crescimento percentual médio (7,6%) na mortalidade por DIC, caracteriza-se por ser o estado com o menor PIB *per capita* – R$ 400,97 – e o segundo estado com maior taxa de analfabetismo entre pessoas com 18 anos de idade ou mais (20,56%), atrás somente de Alagoas (21,47%).^32^

No Rio Grande do Sul, fora evidenciada correlação positiva para índice GINI, escolaridade e distância geográfica – p=0,001 – com a taxa de mortalidade por DIC. A distância geográfica mostra que as distribuições espaciais de centros de referência em intervenção cardiológica influenciam diretamente como fator preditor de óbito, independente, na taxa de mortalidade.^33^ Outro estudo relata ainda que, em consonância às disparidades socioeconômicas, o fator distância geográfica torna-se ainda mais grave. Não obstante, a criação de novos serviços de referência em cardiologia é uma possibilidade para a solução do problema.^34^

O Brasil tem empreendido esforços no sentido de reduzir a ocorrência de DIC. Em 2000, o Ministério da Saúde implantou o Plano de Reorganização da Atenção à Hipertensão Arterial e ao Diabetes Melito. Seus objetivos visam dispor de um sistema informatizado que auxilia no cadastramento e acompanhamento dos portadores de HAS e DM – HiperDia, bem como conhecer a magnitude das doenças, planejar a aquisição de medicamentos e capacitar os profissionais na rede de saúde brasileira para atuar no perfil de atenção à saúde do sistema único de saúde (SUS).^35^

Neste âmbito, estudo realizado em Maringá/PR demonstrou que o impacto das políticas públicas, como a Estratégia de Saúde da Família (ESF), está associado às menores taxas de internação por condições cardiovasculares sensíveis à atenção primária (CCSAP). O sucesso da atenção primária à saúde (APS) é embasado por sua abordagem sobre os fatores de risco para DCV, no suporte ao autogerenciamento da saúde junto ao acompanhamento longitudinal por equipes de saúde, além do empoderamento e da autonomia.^36^

A APS contribui no combate ao tabagismo, na ampliação do acesso aos serviços de saúde e na distribuição de medicamentos para controle dos fatores de risco da DIC.^37^ Em uma pesquisa sobre acesso aos medicamentos pelos usuários da APS, a dimensão de disponibilidade de medicamentos estava baixa, em torno de 46,3% a 64,3%, bem menos que os 80% preconizados pela Organização Mundial da Saúde (OMS).^38^ A alternativa proposta é substituir o medicamento em falta e encaminhar para a Farmácia Popular, embora o controle de doenças crônicas possa vir a ser prejudicado por essa medida.^39^

A tendência de crescimento da mortalidade não ocorre de modo homogêneo entre os estados nordestinos: há crescimento linear em Alagoas e no Rio Grande do Norte, instabilidade das taxas em Pernambuco e um padrão estacionário na maioria dos estados a partir da segunda metade da primeira década do século XXI. Diferentes fatores podem justificar esses achados, tais como as diferenças socioeconômicas intrarregionais, a influência das políticas públicas e a qualidade dos registros de informações.^3,40-43^

Mesmo considerando os cuidados metodológicos adotados, este estudo tem limitações, com destaque para a qualidade dos registros de mortalidade, sendo este um desafio para o adequado monitoramento da situação de saúde e auxílio na tomada de decisão. Adicionalmente, a existência de código *garbage* e as dificuldades operacionais na vigilância do óbito são fatores adicionais que comprometem a qualidade dos registros.

## Conclusão

As taxas de mortalidade na região Nordeste do Brasil apresentaram crescimento significativo em todos os estados que a compõem, sendo maior no Maranhão e no Piauí. O perfil de óbitos caracterizou-se pelo predomínio de homens idosos, raça/cor parda, ensino fundamental ou <4 anos na escola e casados. Durante o período, o sexo masculino demonstrou taxas significativamente maiores que o sexo feminino, ainda que com o mesmo crescimento percentual. As desigualdades nas taxas entre os estados demonstram a necessidade de estratégias consoantes com a realidade e as particularidades locais, e a possível influência das condições de vida da população, sendo essa uma recomendação para estudos futuros. Considerando o achado preocupante de maior taxa de incremento anual na faixa etária de adolescentes, são necessárias maiores investigações sobre a mortalidade cardiovascular nos mesmos.
