# Supplementary material for: Tendência Temporal da Mortalidade por Doenças Isquêmicas do Coração no Nordeste Brasileiro (1996–2016): Uma Análise Segundo Gênero e Faixa Etária
Source: Arq Bras Cardiol. 2021 Jul 15;117(1):51–60. [Article in Portuguese] doi: 10.36660/abc.20200222 (PMC8294739; doi:10.36660/abc.20200222)
Supplement: Translation

# Translation: Temporal Trend of Mortality Due to Ischemic Heart Diseases in Northeastern Brazil (1996–2016): An Analysis According to Gender and Age Group

**Authors:** Gibson Barros de Almeida Santana, Thiago Cavalcanti Leal, João Paulo Silva de Paiva, Leonardo Feitosa da Silva, Lucas Gomes Santos, Tatiana Farias de Oliveira, Rodrigo da Rosa Mesquita, Jéssica Alves Gomes, Carlos Dornels Freire de Souza, Amanda Karine Barros Ferreira Rodrigues

**Affiliations:** 1 BrasilUniversidade Federal de Alagoas, Maceió, AL - Brasil

**Keywords:** Myocardial Ischemia/mortality, Epidemiology, Socioeconomic Factors, Deaths, Statistical Analysis, Demography, Public Health

## Abstract

**Background:**

Ischemic heart disease (IHD) is the leading cause of death among cardiovascular diseases (CVD).

**Objective:**

To describe the sociodemographic profile and analyze the trend in the mortality rate due to IHD, according to sex and by age group, in the states of the Northeast region of Brazil, from 1996 to 2016.

**Methods:**

Ecological study involving IHD mortality in the northeastern states. Variables analyzed: sex, age, education, marital status, ICD-10 category and state of residence. Crude and standardized rates were calculated. Death data were collected from the Mortality Information System (SIM) and population data from the Brazilian Institute of Geography and Statistics (IBGE). In temporal analyzes the regression model by inflection points was used, with the calculation of annual percent change (APC) and average annual percent change of the period (AAPC). A 95% confidence interval and a significance level of 5% were considered.

**Results:**

405916 deaths due to IHD were registered in the northeast region during the study period. The death profile is characterized by men (n=229006; 56,42%), elderly (n=301379; 74,25%), race/color brown (n=197936; 48,76%), elementary or <4 years at school (n=232599; 57,30%) and married (n=179599; 44,25%). There was an unusual highlight to the increase in the annual growth rate in the age group of adolescents (AAPC: 5,2%, p <0.01). The standardized regional mortality rate grew from 30,7 per 100,000 inhabitants in 1996 to 53.8 per 100,000 in 2016 (AAPC 2.8%; p<0.01). All nine states presented a statistically significant growth trend, with emphasis on Maranhão (AAPC 7,6%; p<0.01) and Piauí (AAPC 6,0%; p<0.01).

**Conclusion:**

The prevalent observed profile was male, elderly, race/color brown, low education level and married. Mortality due to IHD presented an upward trend in all states, although with an uneven pattern among the federated units.

## Introduction

Nursing has gained prominence regarding the application of technologies, with opportunities to implement and/or develop them for further evolution of the profession and benefits to the client-professional relationship.^1-3^In this scenario, Chronic Non-Communicable Diseases (CNCDs) - Cardiovascular Diseases (CVD), cancer, chronic respiratory diseases and Diabetes Mellitus (DM) - have gained space in the epidemiological and social context, constituting a global health problem responsible for 40 million deaths annually, 17 million of which as a result of CVD.^4^

In Brazil, in 2016, CNCDs resulted in 707,000 deaths, of which 362,000 were due to CVD.^5^ Due to the continental size of the country, the CNCD mortality dynamics between the regions and units of the Brazilian federation does not show homogeneous features. São Paulo, for example, follows the pattern of developed countries, with a reduction in the mortality rate,^6^ while in the Northeast, under different socioeconomic contexts (worse socioeconomic indicators and difficult access to health), the scenario is the opposite one.^7,8^

Among the CVDs, the most prevalent is Ischemic Heart Disease (IHD), responsible for 116,000 deaths in Brazil in 2016 alone.^5^ IHD presents as insufficient blood flow and inadequate oxygen supply to the heart. This may be the effect of atheromatous plaque rupture, platelet adhesion, thrombosis, and vasospasm, the consequences of which will be ischemic myocardial injury, irreversible damage to cardiomyocytes, and overload hypertrophy in non-necrotic areas.^9^In a pragmatic way, individuals have increased dependence on activities of daily living and mobility, that is, there is a decrease in functional capacity.^10^

The risk factors for these diseases are classified into two categories: modifiable and non-modifiable ones. The modifiable factors are Systemic Arterial Hypertension (SAH), obesity, sedentary lifestyle, inadequate eating habits, smoking, alcohol consumption, dyslipidemias, and insulin resistance; the non-modifiable factors refer to age, gender, ethnicity, and heredity.^11,12^ The control of modifiable risk factors greatly reduces morbidity and mortality from cardiovascular disease.^13^

Research on the temporal evolution of CVD mortality in the Northeast is fundamental for health decision-making, as it can contribute to the definition of priority areas for intervention and the development of strategies and actions aimed at improving the health of the population, especially with regard to risk factors.^14^

The Northeast region lacks descriptive studies on its detailed epidemiological characteristics. Based on the above, this study aimed to describe the sociodemographic profile and analyze the trend of the mortality rate from IHD, according to gender and age group, in the states of the Northeast region of Brazil, from 1996 to 2016. Thus, it broadens the scientific range about their health situation and the social determinants that constitute it.

## Methods

### Data Source and Collection

Death records were obtained from the Mortality Information System (MIS) of the Department of Informatics of the Unified Health System of the Brazilian Ministry of Health (http://www.datasus.gov.br/). During the collection process, the codes I20 to I25 of the International Classification of Diseases (ICD-10) were considered: I20 - Angina pectoris; I21 - Acute myocardial infarction; I22 - Recurrent myocardial infarction; I23 - Some current complications subsequent to acute myocardial infarction; I24 - Other acute ischemic heart disease; I25 - Chronic ischemic heart disease. The population data necessary to calculate the indicators were collected from the 2010 IBGE census.

### Study area

The Northeast region of Brazil comprises nine states (Maranhão, Piauí, Ceará, Rio Grande do Norte, Paraíba, Pernambuco, Alagoas, Sergipe and Bahia) and has an estimated population of 57 million inhabitants, which corresponds to 28% of the national population – being the second most populous region of Brazil.^15^

### Study variables

The study included sociodemographic variables (age group, education, marital status, skin color/ethnicity and states), International Classification of Diseases (ICD-10) category and the crude and standardized mortality rates, according to gender and age group.

To calculate the rates, the following equations were used:

- Annual mortality rate = (Number of deaths from IHD at the site and in the year / Resident population at the site and in the year) x 100,000
- Mortality rate for the period = (Simple average of the number of deaths from IHD at the site in the period / Mid-period resident population at the site) x 100,000

The direct method was adopted for the standardization of mortality rates, considering the Brazilian population of the year 2010 as the standard population. The following age groups were used in the standardization process: 0 to 9 years, 10 to 19 years, 20 to 29 years, 30 to 39 years, 40 to 49 years, 50 to 59 years and 60 years or older.

### Statistical Treatment

Initially, the sociodemographic variables were analyzed using simple descriptive statistics (absolute and relative frequencies). The temporal analysis was performed using the joinpoint regression model. The model analyzes whether a line with multiple segments is better suited to explain the temporal behavior of a data set when compared to a straight line or one with fewer segments. Thus, the trend of each indicator is classified as stationary, increasing or decreasing, according to the slope of the regression line. The Annual Percent Change (APC) and Average Annual Percent Change (AAPC) were calculated.^16^

The following parameters were adopted in the analysis: i) minimum of zero joins, ii) maximum of four joins, iii) model selection by Monte Carlo method (n= 4499 permutations), iv) error autocorrelation method based on date, v) 95% confidence interval (95%CI) and vi) 5% significance level. These analyses were performed with the help of the Joinpoint regression program (version 4.5.0.1, National Cancer Institute, Bethesda, MD, USA).

### Ethical aspects

This study used secondary data from the public domain, in which it is not possible to identify the subjects.

## Results

From 1996 to 2016, 405,916 deaths from IHD were recorded in the Northeast region of Brazil. Of these deaths, 56.42% (n=229006) occurred in males, 74.25% (n=301379) were elderly, 48.76% (n=197936) were brown, 57.30% (n=232599) had elementary school education or <4 years in school, and 44.25% (n=179599) were married. The proportion of blank fields in the variables level of schooling (33.54%), marital status (8.95%) and ethnicity/skin color (15.84%) stood out. As for the cause of death, 85.07% (n=345329) were due to acute myocardial infarction (I21) and 46.37% (n=188217) of the deaths occurred in the states of Pernambuco and Bahia (Table 1).

**Table 1:** – Sociodemographic characterization and ICD-10 category of deaths from ischemic heart disease (IHD) in Northeast Brazil, 1996-2016

Variable	Male n=229006 (56.42%)		Female n=176766 (43.55%)		Unknown n=144 (0.03%)		Total n=405916 (100%)	
	n	%	n	%	n	%	n	%
**Age Range**								
<10 years	72	0.05	49	0.02	0	0.00	121	0.03
10 to 19	469	0.20	215	0.12	1	0.70	685	0.17
20 to 29	2197	0.96	815	0.46	0	0.00	3012	0.74
30 to 39	7086	3.09	3197	1.81	3	2.08	10286	2.53
40 to 49	19380	8.46	10763	6.09	14	9.72	30157	7.43
50 to 59	38131	16.65	21493	12.16	17	11.81	59641	14.69
60 years or older	161295	70.43	139991	79.20	93	64.58	301379	74.25
Ignored	376	0.16	243	0.14	16	11.11	635	0.16
**Skin color/ ethnicity**								
White	58574	25.58	53120	30.05	10	6.94	111704	27.52
Black	17345	7.57	11975	6.77	1	0.70	29321	7.22
Yellow	1062	0.46	928	0.53	2	1.39	1992	0.49
Brown	115714	50.53	82208	46.51	14	9.72	197936	48.76
Indigenous	385	0.17	301	0.17	0	0.00	686	0.17
Unknown	35926	15.69	28234	15.97	117	81.25	64277	15.84
**Level of schooling**								
Elementary or < 4 years at school	129138	56.39	103426	58.51	35	24.30	232599	57.30
High school	15866	6.93	9088	5.14	4	2.78	24958	6.15
Higher education	8303	3.62	3909	2.21	0	0.00	12212	3.01
Unknown	75699	33.06	60343	34.14	105	72.92	136147	33.54
**Marital status**								
Single	45124	19.7	41989	23.75	21	14.58	87134	21.47
Married	125600	54.85	53953	30.52	46	31.94	179599	44.25
Widowed	26413	11.53	58356	33.01	26	18.06	84795	20.89
Divorced	6930	3.03	3642	2.06	1	0.70	10573	2.60
Other	5254	2.29	2228	1.27	2	1.39	7484	1.84
Unknown	19685	8.60	16598	9.39	48	33.33	36331	8.95
**ICD-10 Category**								
I20	885	0.39	875	0.5	0	0.00	1760	0.43
I21	196621	85.86	148585	84.06	123	85.42	345329	85.07
I22	591	0.25	386	0.21	0	0.00	977	0.25
I23	3	<0.01	0	0.00	0	0.00	3	<0.01
I24	6745	2.95	6320	3.58	7	4.86	13072	3.22
I25	24161	10.55	20600	11.65	14	9.72	44775	11.03
**States**								
Maranhão	21847	9.54	13792	7.8	24	16.67	35663	8.79
Piauí	14539	6.35	9446	5.34	16	11.11	24001	5.91
Ceará	33500	14.63	26623	15.06	26	18.06	60149	14.82
Rio Grande do Norte	17347	7.57	13335	7.54	4	2.78	30686	7.56
Paraíba	18825	8.22	14945	8.45	11	7.64	33781	8.32
Pernambuco	59385	25.93	48909	27.67	34	23.61	108328	26.69
Alagoas	11957	5.22	9034	5.11	2	1.38	20993	5.17
Sergipe	6797	2.97	5624	3.20	5	3.47	12426	3.06
Bahia	44809	19.57	35058	19.83	22	15.28	79889	19.68

*I20- Angina pectoris; I21- Acute myocardial infarction; I22- Subsequent myocardial infarction; I23- Certain current complications following acute myocardial infarction; I24- Other acute ischemic heart diseases; I25- Chronic ischemic heart disease.*

The regional standardized mortality rate increased from 30.7/100,000 inhabitants in 1996 to 53.8/100,000 in 2016 (AAPC 2.8%; 95%CI: 1.9 to 3.7; p<0.01). All nine states showed a significant growth trend, especially the state of Maranhão, of which rate increased from 14.8/100,000 in 1996 to 64.0/100,000 inhabitants in 2016 (AAPC 7.6%; 95%CI: 5.7 to 9.6; p<0.01), followed by Piauí, in which the rate increased from 18.7/100,000 in 1996 to 61.5/100,000 inhabitants in 2016 (AAPC 6.0%; 95%CI: 4.3 to 7.8; p<0.01). Pernambuco stood out with the highest number of temporal segments, with four inflections, five temporal segments and AAPC equal to 1.2% (95%CI: 0.0 to 2.5; p<0.01). The states of Rio Grande do Norte and Alagoas showed a linear trend of mortality growth (2.2% in Rio Grande do Norte and 3.6% in Alagoas) (Figure 1).

**Figure 1:** – Time trend of the standardized mortality rate from Ischemic Heart Disease (IHD), according to state of residence. Northeast Brazil, 1996-2016. APC: Annual Percent Change. AAPC: Average Annual Percent Change. *Confidence Interval values have been suppressed in the illustration for better visualization and understanding of the trend lines.

The mean mortality rate in males (42.05/100,000) was 32% higher than in females (31.6/100,000), although with the same mean variation (AAPC 4.4%; 95%CI: 3.4 to 5.4; p<0.01). The adolescent age group showed a statistically significant increase, greater than in the other age groups (AAPC: 5.2%, p<0.01) (Table 2). All states showed an upward trend in both genders, with Maranhão (AAPC 8.7% for men and 9.3% for women) and Piauí (AAPC 7.9% for men and 8.4% for women) standing out (Table 3).

**Table 2:** – Time trend of crude mortality rate from Ischemic Heart Disease (IHD), according to gender and age group. Northeast, Brazil, 1996-2016

Variable	Mortality Rate/100,000			Trend		
	1996	2016	1996-2016	Time period	APC (95%CI; p value)	AAPC (95%CI; p value)
**Gender**						
Male	25.52	60.88	42.05	1996-2004	3.7% (3.0 to 4.5; p<0.01)	4.4% (3.4 to 5.4; p<0.01)
				2004-2007	10.6% (3.4 to 18.3; p<0.01)	
				2007-2016	3.0% (2.4 to 3.6; p<0.01)	
Female	18.78	44.24	31.69	1996-2003	4.0% (3.1 to 5.0; p<0.01)	4.4% (3.6 to 5.2; p<0.01)
				2003-2007	9.2% (5.5 to 13.1; p<0.01)	
				2007-2016	2.6% (2.0 to 3.2; p<0.01)	
**Age Range**						
0 to 9	0.01	0.10	0.05	-	-	-
10 to 19	0.21	0.39	0.31	1996-2016	5.2% (3.0 to 7.5; p<0.01)	5.2% (3.0 to 7.5; p<0.01)
20 to 29	1.00	1.96	1.42	1996-2016	2.8% (2.0 to 3.6; p<0.01)	2.8% (2.0 to 3.6; p<0.01)
30 to 39	5.79	7.18	6.66	1996-2003	-1.5% (-3.5 to 0.5; p=0.2)	0.9% (-0.8 to 2.5; p=0.4)
				2003-2007	6.8% (-1.1 to 15.4; p=0.3)	
				2007-2016	0.2% (-1.2 to 1.6; p=0.2)	
40 to 49	20.44	26.84	25.38	1996-1998	9.8% (1.1 to 19.2; p<0.01)	1.5% (0.1 to 2.9; p<0.01)
				1998-2001	-4.6% (-12.1 to 3.6; p=0.2)	
				2001-2008	4.4% (3.0 to 5.9; p<0.01)	
				2008-2016	-0.7% (-1.6 to 0.2; p=0.2)	
50 to 59	52.99	78.25	74.40	1996-2008	3.3% (2.7 to 3.9; p<0.01)	1.6% (1.1 to 2.2; p<0.01)
				2008-2016	-0.7% (-1.8 to 0.3; p=0.3)	
60 or older	202.03	382.28	323.15	1996-2003	3.3% (2.2 to 4.3; p<0.01)	3.3% (2.5 to 4.1; p<0.01)
				2003-2007	8.5% (4.6 to 12.6; p<0.01)	
				2007-2016	1.0% (0.4 to 1.7; p<0.01)	
Overall population	22.14	52.40	36.82	1996-2004	4.0% (3.3 to 4.7; p<0.01)	4.4% (3.5 to 5.3; p<0.01)
				2004-2007	10.5% (3.9 to 17.4; p<0.01)	
				2007-2016	2.8% (2.2 to 3.4; p<0.01)	

*APC: Annual Percent Change. AAPC: Average Annual Percent Change.*

**Table 3:** – Average Annual Percent Change (AAPC) of the mortality rate from Ischemic Heart Diseases (IHD), according to gender and state of residence. Northeast, Brazil, 1996-2016

State	Male			Female		
	1996	2016	AAPC (95%CI; p value)	1996	2016	AAPC (95%CI; p value)
Maranhão	12.06	62.75	8.7% (7.0 to 10.5; p<0.01)	6.20	38.89	9.3% (7.1 to 11.7; p<0.01)
Piauí	16.16	75.49	7.9% (4.4 to 11.5; p<0.01)	9.98	45.16	8.4% (7.3 to 9.5; p<0.01)
Ceará	20.65	56.89	5.1% (4.5 to 5.8; p<0.01)	15.55	41.70	5.0% (3.9 to 5.1; p<0.01)
Rio Grande do Norte	39.61	74.56	3.5% (3.1 to 3.9; p<0.01)	27.88	53.24	3.9% (3.4 to 4.4; p<0.01)
Paraíba	20.88	78.08	6.8% (5.5 to 8.1; p<0.01)	15.53	60.00	7.1% (5.2 to 9.1; p<0.01)
Pernambuco	49.59	85.82	2.4% (1.5 to 3.3; p<0.01)	36.93	64.36	2.3% (1.3 to 3.3; p<0.01)
Alagoas	23.73	61.02	4.7% (3.1 to 6.3; p<0.01)	15.95	47.96	5.3% (4.5 to 6.1; p<0.01)
Sergipe	19.25	42.47	4.4% (3.1 to 5.7; p<0.01)	18.58	35.80	2.7% (0.5 to 4.9; p<0.01)
Bahia	21.35	39.39	3.6% (3.2 to 4.1; p<0.01)	16.32	29.05	3.6% (2.7 to 4.4; p<0.01)

*APC: Annual Percent Change. AAPC: Average Annual Percent Change.*

In the stratification according to the age group, the regression model showed a tendency for mortality growth in all age segments, with the exception of the group aged 30 to 39 years (AAPC 0.9%; 95%CI: -0.8 to 2.5; p=0.4) (Table 2). The regional mortality rate in individuals aged 60 years or older increased from 202.0/100,000 in 1996 to 382.3/100,000 inhabitants in 2016, with an average annual growth of 3.3% (95%CI: 2.5 to 4.1; p<0.01). The age group 60 years or older was the only one with a statistically significant growth in all states, especially the states of Maranhão (AAPC 8.4%; 95%CI: 6.3 to 10.5; p<0.01) and Piauí (AAPC 6.5%; 95%CI: 5.3 to 7.7; p<0.01) (Table 4).

**Table 4:** – Average Annual Percent Change (AAPC) of crude mortality rate from Ischemic Heart Disease (IHD), according to age range and state of residence. Northeast, Brazil, 1996-2016

**Age Range**	**Maranhão**			**Piauí**			**Ceará**		
	**Rates**		**AAPC**	**Rates**		**AAPC**	**Rates**		**AAPC**
	**1996**	**2016**	**(95%CI)**	**1996**	**2016**	**(95%CI)**	**1996**	**2016**	**(95%CI)**
0 to 9	0.00	0.14	-	0.00	0.19	-	0.00	0.07	-
10 to 19	0.00	0.50	-	0.15	0.84	7.0% (2.9 to 11.3; p<0.01)	0.51	0.56	4.9% (-0.3 to 10.4; p=0.5)
20 to 29	0.12	3.25	9.0% (4.9 to 13.2; p<0.01)	0.93	3.42	6.2% (3.6 to 8.9; p<0.01)	1.16	1.39	0.7% (-0.6 to 2.1; p=0.3)
30 to 39	3.17	11.62	5.8% (4.7 to 7.0; p<0.01)	6.09	10.00	3.5% (2.0 to 5.1; p<0.01)	5.80	5.31	0.8% (-0.3 to 1.9; p=0.3)
40 to 49	10.46	35.96	6.1% (1.5 to 11.0; p<0.01)	14.71	31.10	3.5% (2.5 to 4.5; p<0.01)	15.29	20.40	2.1% (1.2 to 3.1; p<0.01)
50 to 59	29.12	90.17	5.7% (3.2 to 8.1; p<0.01)	34.61	87.61	3.5% (1.4 to 5.6; p<0.01)	38.07	66.06	1.9% (0.5 to 3.4; p<0.01)
60 +	94.19	446.37	8.4% (6.3 to 10.5; p<0.01)	114.40	432.41	6.5% (5.3 to 7.7; p<0.01)	157.12	361.26	3.9% (2.8 to 5.1; p<0.01)
**Age Range**	**Rio Grande do Norte**			**Paraíba**			**Pernambuco**		
	**Rates**		**AAPC**	**Rates**		**AAPC**	**Rates**		**AAPC**
	**1996**	**2016**	**(95%CI)**	**1996**	**2016**	**(95%CI)**	**1996**	**2016**	**(95%CI)**
0 to 9	0.18	0.00	-	0.00	0.16	-	0.00	0.07	-
10 to 19	0.34	0.34	-	0.51	0.15	-	0.23	0.54	4.5% (0.9 to 8.3; p<0.01)
20 to 29	1.14	1.83	4.7% (0.8 to 8.7; p<0.01)	0.92	2.42	5.1% (2.7 to 7.5; p<0.01)	2.10	2.75	1.5% (-0.3 to 3.2; p=0.4)
30 to 39	7.16	7.45	2.0% (0.2 to 3.7; p<0.01)	4.12	7.30	4.7% (3.5 to 5.9; p<0.01)	9.65	9.13	-1.1% (-5.9 to 4.1; p=0.4)
40 to 49	23.52	28.02	1.1% (0.2 to 2.0; p<0.01)	14.54	34.14	4.2% (1.4 to 7.1; p<0.01)	33.08	35.25	-0.2% (-3.3 to 3.0; p=0.2)
50 to 59	69.14	88.46	1.3% (0.4 to 2.2; p<0.01)	31.84	84.49	5.7% (3.9 to 7.4; p<0.01)	98.89	114.28	0.6% (-0.8 to 2.0; p=0.2)
60 +	298.31	452.97	2.4% (1.8 to 3.0; p<0.01)	138.19	469.44	6.3% (4.8 to 7.7; p<0.01)	378.85	520.30	1.2% (0.4 to 1.9; p<0.01)
**Age Range**	**Alagoas**			**Sergipe**			**Bahia**		
	**Rates**		**AAPC**	**Rates**		**AAPC**	**Rates**		**AAPC**
	**1996**	**2016**	**(95%CI)**	**1996**	**2016**	**(95%CI)**	**1996**	**2016**	**(95%CI)**
0 to 9	0.00	0.17	-	0.00	0.00	-	0.00	0.08	-
10 to 19	0.00	0.15	-	0.00	0.00	-	0.13	0.23	-
20 to 29	0.66	2.13	3.2% (0.7 to 5.7; p<0.01)	0.35	0.50	-2.9% (-7.1 to 1.5; p=0.4)	0.75	0.99	0.8% (-0.7 to 2.3; p=0.6)
30 to 39	6.73	5.34	-0.8% (-2.3 to 0.6; p=0.2)	5.59	4.21	1.1% (-0.9 to 3.1; p=0.3)	4.31	5.34	0.4% (-0.5 to 1.2; p=0.6)
40 to 49	31.05	36.91	0.3% (-2.2 to 2.9; p=0.2)	12.47	26.10	2.9% (-0.3 to 6.1; p=0.3)	20.10	16.87	-0.1% (-0.8 to 0.6; p=0.7)
50 to 59	61.26	95.11	1.6% (-1.6 to 4.8; p=0.3)	55.05	67.15	1.6% (0.6 to 2.7; p<0.01)	45.72	50.89	0.3% (-0.7 to 1.3; p=0.2)
60 +	182.72	428.16	4.3% (3.8 to 4.9; p<0.01)	177.36	308.36	2.9% (1.7 to 4.0; p<0.01)	177.50	236.80	2.2% (1.7 to 2.7; p<0.01)

*APC: Annual Percent Change. AAPC: Average Annual Percent Change.*

Finally, a divergence in the trend by age group was observed when comparing the states. In Piauí, for example, there was an increase in all age groups starting from ten years of age, while in Bahia, the increase was observed only in the elderly segment (60 years or older) (Table 4).

## Discussion

This study analyzed the socio-demographic profile and the temporal behavior of mortality from IHD in the Northeast region of Brazil from 1996 to 2016. The profile of deaths was characterized by a predominance of males, involvement of the elderly, brown ethnicity/skin color, and low level of schooling. The temporal analysis showed an increase in the mortality rate in the region and in all states, especially in Maranhão and Piauí.

The profile observed in this study is in line with that observed in the literature.^1,7,17-20^ The population’s aging process brings with it an increase in risk factors for cardiovascular diseases, especially dyslipidemia, obesity, and SAH.^2^ A study conducted in the city of São Paulo shows that the odds ratio of developing CVD is higher in diabetics (Odds Ratio - OR 1.90), smokers (OR 1.49), overweight individuals (OR 1.57), and hypertensive individuals (OR 2.22).^20^ It is estimated that the prevalence of SAH in the elderly is six to eight times higher than in young adults, justified by poor blood pressure control, due to the chronicity of the disease and sensitivity and restrictions in pharmacotherapy. In a study carried out in Goiânia, state of Goiás, of the 912 individuals with SAH that were interviewed, 72.6% were undergoing treatment and only 50.8% had achieved blood pressure control.^21^

It is pertinent to highlight the growth of mortality in adolescence, as observed in this investigation. In this population, studies point to a wide range of factors associated with overweight and obesity, such as i) excessive intake of simple sugars and fats, ii) insufficient consumption of fruits and vegetables, and iii) sedentary lifestyle, which may result in early mortality.^22^ The Study of Cardiovascular Risks in Adolescents (ERICA) carried out from 2013 to 2014 reported the prevalence of metabolic syndrome in 3.3% of those who were overweight and 21.7% of those who were obese in the Northeast region.^23^

The medical importance of lipid profile alterations is related to the presence of subclinical atherosclerosis and the possibility of predicting dyslipidemia in adulthood; in the ERICA study, we observed a higher prevalence of low HDL (High Density Lipoprotein) levels in the North and Northeast regions of Brazil.^24^ A study conducted in Belém, state of Pará in 2006, involving 437 children and adolescents, showed that 28.8% were overweight and 36% had high body fat percentage. Of these, 49% showed changes in lipid profile, especially low HDL levels.^25^ Similar results were observed in Porto Alegre, state of Rio Grande do Sul, where an increase in dyslipidemia secondary to obesity was observed, characterized by low HDL levels and increased triglyceride levels, secondary to insulin resistance.^26^

The cardiovascular risk is also influenced by gender, being higher in the male population. A study on hypertension in Goiânia - GO, conducted in 2010, showed that the rate of control of blood pressure levels is lower in the male population (44.0% in men and 54.8% in women).^21^ In Montes Claros, state of Minas Gerais, 62.8% of diabetes mellitus cases are identified in women, justified by the greater demand for medical care by this population. Women also show greater and better adherence to treatment.^27^ Moreover, women have biological and behavioral characteristics capable of reducing the risk of cardiovascular disease, highlighting the protective role of estradiol on the vascular endothelium, greater access to health services and better performance in the control of risk factors.^17^

The influence of socioeconomic development and access to health services on the pattern of mortality has been evidenced in different studies.^28-30^ Research conducted in 2012 in the states of Rio de Janeiro, Rio Grande do Sul, and São Paulo showed a correlation between increased *per capita *GDP and educational accomplishments with decreased mortality from IHD.^31^ In the Northeast, Maranhão, at the same time that it showed the highest average percentage growth (7.6%) in mortality from IHD, it is characterized by being the state with the lowest *per capita* GDP - R$400.97 - and the second state with the highest illiteracy rate among people aged 18 or over (20.56%), second only to the state of Alagoas (21.47%).^32^

In Rio Grande do Sul, a statistically significant positive correlation was found for the GINI index and education, in addition to geographic distance. The distance shows that the geographical distributions of cardiac intervention reference centers directly influence, as an independent predictor of death, the mortality rate from IHD.^33^ Another study also reports that, in line with socioeconomic disparities, the geographic distance factor becomes even more serious. Nevertheless, the creation of new reference services in cardiology is a possibility to solve the problem.^34^

Brazil has undertaken efforts to reduce the occurrence of IHD. In 2000, the Ministry of Health implemented the Reorganization Care Plan for Hypertension and Diabetes Mellitus. Its objectives are to have a computerized system to help with the registration and follow-up of patients with SAH and DM – HiperDia -, as well as to know the magnitude of the diseases, plan the acquisition of medicines, and train professionals in the Brazilian health network to act in the health care profile of the unified health system (UHS).^35^

In this context, a study conducted in Maringá, state of Paraná, showed that the impact of public policies, such as the Family Health Strategy (FHS), are associated with lower rates of hospitalization from Cardiovascular Conditions Sensitive to Primary Care (CCSPC). The success of the Primary Health Care (PHC) strategy is based on its approach to CVD risk factors, on the support to the self-management of health, along with longitudinal monitoring by health teams, in addition to their empowerment and autonomy.^36^

PHC contributes to the fight against smoking, to the expansion of access to health services, and to the distribution of medicines to control the IHD risk factors.^37^ In a survey regarding the access to medication by PHC users, the medication availability dimension was low, around 46.3% to 64.3%, well below the 80% recommended by the World Health Organization (WHO).^38^ The proposed alternative is to replace the non-available drug and refer patients to the “People’s Pharmacy”, although the control of chronic diseases may be jeopardized by this measure.^39^

The mortality growth trend is not homogeneous among the Northeastern states: there is a linear growth in the states of Alagoas and Rio Grande do Norte, rate instability in Pernambuco, and a stationary pattern in most states from the second half of the first decade of the 21^st^ century. Different factors may justify these findings, such as intra-regional socioeconomic differences, the influence of public policies, and the quality of information records.^3,40-43^

Even when considering the adopted methodological care, this study has limitations, especially the quality of mortality records, which is a challenge for the adequate monitoring of the health status and aid in decision-making. Additionally, the existence of garbage codes and operational difficulties in death surveillance are additional factors that compromise the quality of the records.

## Conclusion

The mortality rates in the Northeast region of Brazil showed significant growth in all its states, being higher in Maranhão and Piauí. The profile of deaths was characterized by a predominance of elderly men, mixed race ethnicity/skin color, elementary school or <4 years in school, and married individuals. During the period, the male gender showed significantly higher rates than the female gender, although with the same percentage of growth. The rate inequalities between the states demonstrate the need for strategies consistent with the local reality and particularities and the possible influence of living conditions of the population, with this being a recommendation for future studies. Considering the finding of a higher annual rate of increase in the adolescent age group, which is a matter of concern, further investigations on cardiovascular mortality in adolescents are required.
